# Attitudes and Knowledge Regarding the Therapeutic Use of Cannabinoids among Community Pharmacists: A Pilot Cross-Sectional Study in Amman, Jordan

**DOI:** 10.3390/healthcare11050694

**Published:** 2023-02-26

**Authors:** Firas H. Bazzari, Amjad H. Bazzari

**Affiliations:** 1 Faculty of Pharmacy, Jerash University, Jerash 26150, Jordan; 2 Department of Basic Scientific Sciences, Faculty of Arts & Sciences, Applied Science Private University, Amman 11931, Jordan

**Keywords:** pharmacy practice, medicinal cannabis, cannabinoids, cannabinoid-derived drugs, pharmacology, community pharmacy

## Abstract

There is an increasing interest in the therapeutic use of cannabis worldwide, with a number of cannabinoid-derived drugs currently approved by the Food and Drug Administration (FDA) for certain indications. This study was conducted via a printed questionnaire and aimed to explore the attitudes and knowledge regarding the therapeutic use of cannabis and cannabinoids among community pharmacists residing in Amman, Jordan. The results revealed a neutral to low agreement level regarding the medical usefulness of cannabis; however, a higher agreement level was observed for FDA-approved cannabinoid-derived drugs. The majority of the participants reported that they did not learn enough regarding cannabinoids, do not adequately remember what they have learned, and do not actively look for information after graduation. The average percentages of correct identification of cannabis/cannabinoid FDA-approved drug indications, common adverse effects, interacting drugs, and cautions/contraindications were 40.6%, 53%, 49.4%, and 57.3%, respectively, with an overall correct identification rate of 51.1% of the participants. In conclusion, the results indicate an inadequate level of knowledge with a significant room for improvement regarding the various aspects of cannabinoid pharmacology.

## 1. Introduction

Cannabinoids are naturally derived compounds from *Cannabis sativa*, family *Cannabaceae*, which has been cultivated by mankind since old times for medical applications [1]. The recorded history of cannabis use dates back to ancient China and was pharmacologically described as a medicine in Chinese early medical books for a number of uses, such as chronic pain associated with rheumatoid arthritis, gastrointestinal-related disorders, gynecologic pain conditions, and malaria among multiple other uses [2]. The therapeutic use of cannabis flourished in the west in the late 19th and early 20th century. However, the active constituents of the plant were yet to be determined at the time and the first active compound, tetrahydrocannabinol (THC), was later isolated in 1964 [3]. This delay has led to the displacement of cannabis with other medications, such as aspirin, for their well-defined safety and efficacy profiles when compared to the variations of cannabis extracts [3].

Cannabis was brought back to the picture as a potential therapeutic candidate following the discovery of the endocannabinoid system, which consists of endogenous cannabinoids, such as anandamide and 2-arachidonoyl-sn-glycerol (2-AG), and cannabinoid receptors (i.e., CB1 and CB2) [4]. Endocannabinoids are found to play a role in tuning synaptic function and, in turn, influence a variety of physiological and behavioral processes [5]. This is in addition to a multitude of central effects attributed to the use of cannabis [6]. Extensive scientific research efforts have warranted Food and Drug Administration (FDA) approval for a number of cannabis-derived and synthetic cannabis-related drug products for selected indications [7]. Currently, Epidiolex^®^ (a purified cannabidiol) is approved for the use in patients ≥2 years old with Lennox–Gastaut syndrome and Dravet syndrome and for patients ≥1-year old with tuberous sclerosis complexes [8]. In addition, Marinol^®^ and Syndros^®^, both containing dronabinol (a synthetic derivative of THC), are approved for nausea associated with cancer chemotherapy and anorexia associated with weight loss in acquired immunodeficiency syndrome (AIDS) patients [9]. Lastly, Cesamet^®^, which contains nabilone (a synthetic derivative of THC), is approved for the nausea associated with cancer chemotherapy [10]. On the other hand, the FDA is aware of the use of multiple unapproved cannabis and/or unapproved cannabis-derived products for the management of certain conditions and, thereby, the FDA highlights a caveat regarding the safety and effectiveness of unapproved compounds [7]. In contrast, the European Medicines Agency (EMA) only authorized the use of Epidiolex^®^ (cannabidiol) and for the same indications approved by the FDA [11].

Pharmacists are primary healthcare providers with an integral role in medication stewardship. In some countries, pharmacists are the point of access to medicinal cannabis and the main reference for patients and prescribers regarding the proper medical use of cannabis [12]. The use of medicinal cannabis is not permitted and no FDA-approved cannabinoid-based medication is currently available in Jordan, which is similar to other Middle East countries (except for Lebanon) [13]. While no specific number for pharmacists is available, recent reports by the ministry of foreign affairs and expatriates show that there is well over a million Jordanian citizens (around 10% of the total population) living abroad [14]. In addition, a total of 18,373 international students from 64 different countries are currently enrolled in Jordanian universities, 19.9% of whom are medical and health-related students, according to the 2019/2020 annual report by the ministry of higher education and scientific research [15]. From a national point of view, the Jordan FDA (JFDA) is the official body responsible for the registration of new drugs in the country and among the basic requirements is the approval and registration of the drug in the country of origin [16]. These findings and the unique position of pharmacists as drug experts necessitates them to be knowledgeable enough regarding herbal and synthetic pharmacologically active compounds, even with unavailable status as for cannabinoids, to ensure patient safety [17]. This entails the ability to evaluate approved drug indications, contraindications, interactions, and adverse events through an evidence-based approach.

This pilot study aims to assess the attitudes and knowledge regarding the therapeutic use of cannabinoids and cannabinoid-derived drugs among community pharmacists in Amman, Jordan, in order to identify areas for improvement in pharmacy program curricula and/or continued education to ensure that community pharmacists are equipped with adequate and accurate knowledge to provide patient education regarding the medical use and harms of cannabis and cannabinoid-based drugs if required.

## 2. Materials and Methods

### 2.1. Sample

The pilot sample size was determined as described by Viechtbauer et al. [18] using the following equation:

$$n=\frac{\ln\left(1-\gamma \right)}{\ln\left(1-\pi \right)}$$

The required sample was 59, confidence interval 95% (*γ* = 0.95) with probability (*π* = 0.05), and a total of 60 participants were included in the study.

### 2.2. Protocol and Ethical Considerations

The pilot survey was conducted using a printed questionnaire in English, the official teaching language for pharmacy programs in Jordan, and distributed by the researchers among community pharmacists residing in Amman during working hours. An explanation of the study aims was provided prior to handing the questionnaire. The participants were informed that no personal identifying information will be asked and the collected data will be solely used for scientific research purposes. The participation was voluntary, and the participants were not paid or compensated.

The study was approved by the department of pharmacy council at Jerash university (approval number: 2/أ/2022, date: 21 November 2022), and was conducted with strict adherence to the guidelines of the declaration of Helsinki regarding anonymity, voluntary participation, and data protection [19].

### 2.3. Questionnaire

The study questionnaire included three main domains: (1) demographics, (2) attitudes (i.e., personal opinions), and (3) knowledge.

The demographics domain included questions regarding the age, gender, graduation date, years of community pharmacy practice, grade point average (GPA) rating, and educational level (i.e., if the participants hold any postgraduate degree).

The attitudes domain included a total of 6 questions; (1) “Do you think that cannabis has medical usefulness?”, (2) “Do you think cannabis-derived FDA-approved drugs are useful?”, (3) “Do you think medicinal cannabinoid benefits outweigh their risks?”, (4) “Do you think you learned enough about medicinal cannabinoids during your undergraduate studies?”, (5) “Do you think you adequately remember what you learned?”, and (6) “Do you think you are knowledgeable enough to educate patients on the adverse effects, interactions and contraindications of cannabinoids?”. The responses to the previous questions were based on a 5-point Likert agreement scale; (1) “To a very high degree”, (2) “To a high degree”, (3) “Moderate”, (4) “To a low degree”, and (5) “To a very low degree”.

In the knowledge domain, the participants were initially asked whether they learned about cannabis and cannabinoids after graduation to account for any influence of self-learning, if yes, the participants were then asked if they purposely sought the information or if it was by chance and which sources of information they used (i.e., books, scientific articles, news, or the Internet). Then, the participants were asked “What central pharmacological effects do cannabis or cannabinoids have?”, to assess their general perceptions of central cannabinoid effects, and given the following options “Hallucinogenic”, “Depressant”, “Stimulant”, “All answers”, or “None of the answers”. Lastly, the participants were asked to answer 4 checkbox questions regarding the indications of FDA-approved cannabinoid-derived drugs as well as cannabis/cannabinoids adverse effects/events, moderate/major drug interactions, and contraindications. The aforementioned questions were developed based on the discussion of scientific literature and reputable sources [7,20,21,22,23,24,25,26,27,28,29].

### 2.4. Statistical Analysis

The data analysis was conducted using JASP software (Version 0.16.2, www.jasp-stats.org). All the results are presented as mean ± standard deviation (±SD) or as counts (n) and percentages (%). The gender-based differences between the participant demographic variables were assessed using either t-test for parametric data (age and years of experience) or Chi-square test for non-parametric data (education and GPA rating). The distribution of the participant responses to attitudes questions, based on a 5-point Likert agreement scale, and knowledge questions were compared across genders and GPA ratings using Chi-square test. To assess the influence of age and experience on participant attitudes, the responses were coded into ordinal variables and Spearman’s rank correlation test was conducted. The reliability of the attitudes questions was assessed using the single-test reliability analysis, and the Cronbach’s α was calculated. For all the statistical tests, a *p* value less than 0.05 was considered significant (*).

## 3. Results

### 3.1. Participant Demographics

The study included the responses of a total of 60 on-duty community pharmacists residing in Amman, Jordan, from both genders: males (*n* = 18, 30%) and females (*n* = 42, 70%). All 60 participants completed their undergraduate degree in pharmacy from universities in Jordan and all completed the questionnaire; thus, no responses were excluded. The mean age of all the participants was 30.48 years (SD ± 8.6), ranging from 22 to 59 years of age with from 1 to 38 years of experience in community pharmacy (6.37 ± 7.53 years). The mean age, and accordingly years of experience, was higher for males (35.06 ± 10.95 years of age) compared to females (28.52 ± 6.6, *p* < 0.05). The vast majority of participants (95%) had a bachelor level of education while three participants (5%) were master’s degree holders. The self-reported undergraduate GPA rating was collected as well. The number of participants reporting an Excellent, Very Good, Good, and Satisfactory GPA rating was 8 (13.3%), 22 (36.7%), 26 (43.3%), and 4 (6.7%), respectively. The GPA rating variable was dependent on gender (χ2 = 10.7, *p* < 0.05) with a higher percentage of females exhibiting an Excellent GPA rating (16.67%) and a lower percentage with a Satisfactory rating (0%) compared to the male participants (5.56% and 22.2%, respectively). The collected participant demographics are summarized in Table 1.

### 3.2. Attitudes towards Medicinal Cannabinoids

The attitudes of the participants toward the medical usefulness of and their education regarding medicinal cannabinoids were assessed using six questions based on 5-point Likert agreement scale ranging between “To a very high degree” and “To a very low degree”. The internal reliability of these questions was adequate with a Cronbach’s α of 0.71 (95% confidence interval: 0.57–0.81). The attitudes questions and participant responses are summarized in Table 2. When asked about their opinion on whether cannabis has medicinal usefulness, most participants (36.7%) were neutral with a “Moderate” response, followed by “Low” agreement (25%), “High” agreement (20%), “Very low” agreement (15%), and lastly “Very high” agreement (3.3%). The response distribution of this question was dependent on gender (χ2 = 15.2, *p* < 0.01) as most male participants (61.1%) were in disagreement while most females (45.2%) were neutral. However, the agreement rate was similar between genders (22.2% and 23.8% for males and females, respectively). The participant agreement rate was higher (35%) when asked regarding the medicinal usefulness of cannabis-derived FDA-approved drugs while the majority (41.7%) were neutral. In contrast to medicinal cannabis, most males were in agreement with FDA-approved cannabinoid medications (44.4%) while most females (47.6%) were neutral; thus, the responses to this question were gender-dependent (χ2= 12.6, *p* < 0.01). On the other hand, gender did not influence the third question responses on whether the benefits of medicinal cannabinoids outweigh their risks (χ2 = 6.08, *p* > 0.05) as the majority (50%) of participants were in disagreement including both males (44.4%) and females (52.4%), while only 15% agreed. Regarding their personal opinions on their undergraduate education about medicinal cannabinoids, most participants do not think they learned enough (53.3%), adequately remember what they learned (50%), or are knowledgeable enough to educate patients on corresponding adverse effects, interactions, and contraindications (46.7%). A summary of participant attitude responses can be found in Table 2. The distribution of participant responses to all six attitudes questions was independent from the GPA rating (*p* > 0.05) and did not correlate with either age or years of experience (*p* > 0.05). The impact of participant demographics on the attitude responses is summarized in Table 3.

### 3.3. Knowledge of Medicinal Cannabinoids

Following the collection of participant demographics and attitudes, the third part of the questionnaire focused on assessing the knowledge of participating community pharmacists regarding the pharmacology of medicinal cannabinoids. In order to control for the potential influence of self-learning, the participants were first asked whether they learned about cannabis or medicinal cannabinoids following the completion of their undergraduate studies, if it was by chance or out of self-interest and which sources they sought the information from. One third of the participants (*n* = 20) reported learning about cannabinoids following graduation, of whom 14 participants (23.3% of total) actively sought the information; however, the most-sought source of information was the Internet (*n* = 9), followed by scientific articles (*n* = 7) and books (*n* = 4); therefore, none of the participants received formal postgraduate education on medicinal cannabinoids. Then, the participants were asked about the central pharmacological effects of cannabis and given the following options: “Hallucinogenic”, “Depressant”, “Stimulant”, “All answers”, and “None of the answers”. Most participants answered with “Hallucinogenic” (40%), followed by “All” (25%), “Stimulant” (20%), “Depressant” (11.7%), and “None” (3.3%). As summarized in Table 4, the distribution of answers to this question was independent from gender (χ2 = 1.53, *p* > 0.05) and the self-reported GPA rating (χ2 = 7.13, *p* > 0.05) and was very similar between the participants who did not learn about cannabis or medicinal cannabinoids following graduation and those who reported learning (χ2 = 3.08, *p* > 0.05).

The participants were then assessed regarding four key pharmacological aspects of medicinal cannabis and cannabinoids: FDA-approved indications, adverse effects, drug interactions, and cautions/contraindications, Table 5. For each of these categories, the participants were presented with options that included correct and incorrect answers and asked to identify the correct ones.

The options were provided for each pharmacology question in no particular order and each participant was allowed to choose one, multiple, or all answers. The average percentages of the correct identification of cannabis/cannabinoid FDA-approved drug indications, common adverse effects, interacting drugs, and cautions/contraindications were 40.6%, 53%, 49.4%, and 57.3%, respectively, with an overall correct identification rate of 51.1% of the participants. On the other hand, the percentages of identifying an incorrect indication, adverse effect, interacting drug, and caution/contraindication were 44.6%, 24%, 20.4%, and 22.2%, respectively, with an overall incorrect identification rate of 27.9% of the participants. Interestingly, out of a total of 35 options, the identification of only 3 was dependent on gender, which were incorrect options, and of only 2 was dependent on the self-reported participant GPA rating, 1 of which was incorrect. The category with the lowest correct and also the highest incorrect identification rate was the FDA-approved indications of cannabinoid-derived drugs.

## 4. Discussion

This pilot study is the first to explore the attitudes and knowledge regarding the therapeutic use of cannabinoids among community pharmacists in Amman, Jordan. The overall results show a low level of knowledge with a significant room for improvement in terms of cannabinoid pharmacology.

The sample was consistent with the general distribution of community pharmacists in Jordan in terms of gender with a total of 64.26% females and 35.74% males as highlighted in the latest report by the Jordan Pharmacists Association (JPA) [30]. In addition, the sample included a wide range of participants’ age (from 22 to 59 years old) and experience (from 1 to 38 years). Moreover, the participants’ GPA was distributed among all categories. Nevertheless, neither the GPA nor years of experience of the participants had any significant influence on their responses. Furthermore, the majority of the participants reported that they did not learn enough regarding cannabinoids, do not adequately remember what they have learned, and do not actively look for information after graduation. All combined, these factors can be considered the major contributors to the current status of pharmacological knowledge regarding the proper medical use of medicinal cannabis and cannabinoid-derived drugs. However, from a different perspective, it can be argued and justified that there is no actual need in this regard since there are no approved cannabinoid-derived drugs in Jordan.

On the other hand, the recreational use of cannabis is another major factor to be considered. In countries where cannabis is legalized, recreational use was found to be dominant when compared to medical use [31]. Consequently, calls are rising for public education and awareness regarding the harms and adverse effects of cannabis, especially given that the legalization of cannabis is generally perceived as risk free [32]. Furthermore, the abuse of illicit synthetic cannabinoids is also rising and is considered a global health concern [33]. A recent study by AbuAlSamen et al., conducted to assess the knowledge and perceptions of illicit synthetic cannabinoids among Jordanian university students, revealed the poor knowledge of the students about illicit synthetic cannabinoids; nevertheless, the vast majority of the students considered the use of illicit synthetic cannabinoids to be ethically unacceptable and associated with detrimental health effects [34]. These findings are consistent with the careful attitude observed among the sample regarding the medical use of cannabinoids.

Despite that a minor fraction of participants reported looking for information regarding cannabinoids, the majority of them used the Internet as their primary sought source. On the positive side, online platforms and social media can be utilized by official bodies for spreading awareness and public education regarding the proper use and harms of certain medicines [35]. Nevertheless, due to the limitless freedom offered by the Internet with the ability to access and share virtually anything, the accuracy of medical information remains a concern [36]. Accordingly, the Internet can be a “dangerous” source of knowledge, since there is much news that is unofficial or incorrect. For instance, anecdotal evidence suggests that medicinal cannabis may benefit the management of chronic neuropathic and cancer-associated pain [37]; however, robust investigations either proved the opposite or highlighted the lack of conclusive evidence [38,39]. Such claims and many other examples can sometimes be inflated by certain online news outlets and social media platforms, therefore providing inaccurate description and, in turn, creating misconceptions among the public [40,41]. Therefore, continued education courses, conferences, or webinars can be considered for improving knowledge. All in all, pharmacists should always follow an evidence-based approach and be aware of such issues to ensure patient safety.

In comparison to other populations, similar patterns can be found in the literature. For instance, studies of several states in the USA with implemented cannabis programs have revealed that, even after years of program implementation, there is still a significant need for further pharmacists training and education regarding various regulatory and clinical aspects of cannabinoids [42,43]. Furthermore, studies conducted in Australia, Canada, European countries, and Lebanon, in addition to others, have also highlighted the need for further cannabinoid education and training for pharmacists and other healthcare professionals [44,45,46,47]. On the other hand, numerous studies assessing the knowledge among pharmacy students also revealed a gap in pharmacy teaching curricula, as students were found to be neither knowledgeable enough nor confident to council patients regarding cannabinoids. These findings further necessitate the importance of incorporating competency-based courses in order to address this gap and ensure optimal patient care [48,49,50,51]. Therefore, the implementation of both under- and post-graduate courses and training are key factors to be considered.

## 5. Conclusions

In conclusion, community pharmacists in this sample have a low and neutral agreement level regarding the therapeutic usefulness of cannabis and FDA-approved cannabinoid-derived drugs, respectively. In addition, the current results have demonstrated the inadequacy of the pharmacological knowledge of community pharmacists in Jordan regarding cannabis and cannabinoid-derived drugs. This is true across all aspects of cannabinoid pharmacology including their indications, side effects, interactions, and contraindications. The results, therefore, highlight the need for improvements in undergraduate and/or continued education in this regard.

## Figures and Tables

**Table 1:** Participant demographics.

Variable	All	Males	Females	*p* Value
Age in years: mean (SD)	30.48 (8.6)	35.06 (10.95)	28.52 (6.6)	0.006 ^a,^*
Experience: mean (SD)	6.37 (7.53)	10.17 (10.29)	4.74 (5.36)	0.009 ^a,^*
Education: count (%)				0.897 ^b^
Bachelor	57 (95%)	17 (94.4%)	40 (95.2%)	
Masters	3 (5%)	1 (5.6%)	2 (4.8%)	
GPA Rating: count (%)				0.013 ^b,^*
Excellent	8 (13.3%)	1 (5.6%)	7 (16.7%)	
Very Good	22 (36.7%)	6 (33.3%)	16 (38.1%)	
Good	26 (43.3%)	7 (38.9%)	19 (45.2%)	
Satisfactory	4 (6.7%)	4 (22.2%)	0 (0%)	

* Significant difference between genders (*p* < 0.05), ^a^ *t*-test, and ^b^ Chi-square test.

**Table 2:** Participant attitudes towards medicinal cannabinoid usefulness and education.

Questions	Participant Agreement Level, Count (%)				
	Very Low	Low	Moderate	High	Very High
Do you think that cannabis has medical usefulness?	9 (15%)	15 (25%)	22 (36.7%)	12 (20%)	2 (3.3%)
Do you think cannabis-derived FDA-approved drugs are useful?	6 (10%)	8 (13.3%)	25 (41.7%)	13 (21.7%)	8 (13.3%)
Do you think medicinal cannabinoid benefits outweigh their risks?	10 (16.7%)	20 (33.3%)	21 (35%)	7 (11.7%)	2 (3.3%)
Do you think you learned enough about medicinal cannabinoids during your undergraduate studies?	13 (21.7%)	19 (31.7%)	19 (31.7%)	8 (13.3%)	1 (1.7%)
Do you think you adequately remember what you learned?	7 (11.7%)	23 (38.3%)	18 (30%)	10 (16.7%)	2 (3.3%)
Do you think you are knowledgeable enough to educate patients on the adverse effects, interactions, and contraindications of cannabinoids?	9 (15%)	19 (31.7%)	18 (30%)	9 (15%)	5 (8.3%)

**Table 3:** Impact of demographics on participant attitudes.

Questions	Impact of Demographics, *p* Value			
	Age ^a^	Experience ^a^	Gender ^b^	GPA ^b^
Do you think that cannabis has medical usefulness?	0.316	0.44	0.004 *	0.154
Do you think cannabis-derived FDA-approved drugs are useful?	0.476	0.528	0.016 *	0.84
Do you think medicinal cannabinoid benefits outweigh their risks?	0.08	0.096	0.193	0.275
Do you think you learned enough about medicinal cannabinoids during your undergraduate studies?	0.517	0.473	0.141	0.884
Do you think you adequately remember what you learned?	0.858	0.769	0.454	0.866
Do you think you are knowledgeable enough to educate patients on the adverse events, interactions, and contraindications of cannabinoids?	0.349	0.522	0.318	0.542

* Significant (*p* < 0.05), ^a^ Spearman’s correlation test, and ^b^ Chi-square test.

**Table 4:** Impact of demographics on participant perceptions of central cannabinoid pharmacology.

Answers Count (%) ^a^	What Central Pharmacological Effects Do Cannabis or Cannabinoids Have?					
	Hallucinogenic	Stimulant	Depressant	All	None	*p* Value ^b^
Total	24 (40%)	12 (20%)	7 (11.7%)	15 (25%)	2 (3.33%)	
Gender						0.821
Males	6 (33.3%)	5 (27.8%)	2 (11.1%)	4 (22.2%)	1 (5.6%)	
Females	18 (42.8%)	7 (16.7%)	5 (11.9%)	11 (26.2%)	1 (2.4%)	
GPA rating						0.849
Excellent	5 (62.5%)	1 (12.5%)	1 (12.5%)	1 (12.5%)	0 (0%)	
Very Good	7 (31.8%)	6 (27.3%)	3 (13.6%)	5 (22.7%)	1 (4.6%)	
Good	11 (42.3%)	3 (11.5%)	3 (11.5%)	8 (30.7%)	1 (3.8%)	
Satisfactory	1 (25%)	2 (50%)	0 (0%)	1 (25%)	0 (0%)	
Self-learning after graduation						0.544
Yes	7 (35%)	6 (30%)	1 (5%)	5 (25%)	1 (5%)	
No	17 (42.5%)	6 (15%)	6 (15%)	10 (25%)	1 (2.5%)	

^a^ Within row percentages and ^b^ Chi-square test.

**Table 5:** Participants’ knowledge of medicinal cannabis and cannabinoid pharmacology.

Questions and Answers	Count (%) ^a^	Gender, *p* Value ^b^	GPA, *p* Value ^b^
Which indications are cannabinoid-based drugs approved for by the FDA?			
Seizures in Lennox–Gastaut syndrome ^c^	21 (35.0%)	0.859	0.756
Nausea associated with cancer chemotherapy ^c^	35 (58.3%)	0.391	0.171
Anorexia in AIDS patients ^c^	17 (28.3%)	0.574	0.168
Pain associated with cancer ^i^	40 (66.7%)	0.550	0.226
Chronic neuropathic pain ^i^	31 (51.7%)	0.338	0.933
Resistant major depressive disorder ^i^	25 (41.7%)	0.153	0.214
Weight loss in obese patients ^i^	11 (18.3%)	0.216	0.604
Which adverse effects/events are commonly caused by cannabis/cannabinoids?			
Anxiety ^c^	43 (71.7%)	0.574	0.800
Memory/cognitive impairment ^c^	42 (70.0%)	0.806	0.799
Insomnia ^c^	30 (50.0%)	0.573	0.764
Tachycardia ^c^	31 (51.7%)	0.866	0.995
Orthostatic hypotension ^c^	13 (21.7%)	0.945	0.628
Anemia ^i/u^	9 (15.0%)	0.813	0.444
Loss of appetite ^i/u^	28 (46.7%)	<0.001 *	0.402
Constipation ^i/u^	17 (28.3%)	0.01 *	0.558
Hyperglycemia ^i/u^	5 (8.3%)	0.61	0.727
Glaucoma ^i/u^	13 (21.7%)	0.047 *	0.966
Which drugs have moderate or major interactions with cannabis/cannabinoids?			
Alprazolam ^c^	31 (51.7%)	0.128	0.857
Fluoxetine ^c^	25 (41.7%)	0.391	0.138
Diphenhydramine ^c^	19 (31.7%)	0.672	0.917
Pregabalin ^c^	27 (45.0%)	0.101	0.863
Phenytoin ^c^	33 (55.0%)	0.955	0.635
Warfarin ^c^	43 (71.7%)	0.574	0.629
Amoxicillin ^i^	6 (10.0%)	0.851	0.559
Ibuprofen ^i^	9 (15.0%)	0.581	0.208
Omeprazole ^i^	20 (33.3%)	1.000	0.439
Acetaminophen ^i^	14 (23.3%)	0.424	0.913
Which conditions are cautions/contraindications for cannabis/cannabinoid usage?			
Schizophrenia ^c^	27 (45.0%)	0.533	0.464
Major depressive disorder ^c^	25 (41.7%)	0.391	0.055
Cardiac arrhythmia ^c^	36 (60.0%)	0.301	0.047 *
Cardiovascular disorders ^c^	36 (60.0%)	0.645	0.947
Pregnancy ^c^	48 (80.0%)	0.260	0.569
Glaucoma ^i^	15 (25.0%)	0.329	0.800
Hypothyroidism ^i^	12 (20.0%)	0.673	0.240
Cancer ^i^	13 (21.7%)	0.538	0.034 *

* Significant (*p* < 0.05), ^a^ % of all, ^b^ Chi-square test, ^c^ correct, ^i^ incorrect, and ^u^ uncommon.

## Data Availability

Available on request from the corresponding author, F.H.B.
